# Sustainable–Green Synthesis of Silver Nanoparticles Using Aqueous *Hyssopus officinalis* and *Calendula officinalis* Extracts and Their Antioxidant and Antibacterial Activities

**DOI:** 10.3390/molecules27227700

**Published:** 2022-11-09

**Authors:** Aiste Balciunaitiene, Viktorija Puzeryte, Vitalijs Radenkovs, Inta Krasnova, Patrick B. Memvanga, Pranas Viskelis, Paulina Streimikyte, Jonas Viskelis

**Affiliations:** 1 Lithuanian Research Centre for Agriculture and Forestry, Institute of Horticulture, 54333 Kaunas, Lithuania; 2 Processing and Biochemistry Department, Institute of Horticulture, LV-3701 Dobele, Latvia; 3 Research Laboratory of Biotechnology, Division of Smart Technologies, Latvia University of Life Sciences and Technologies, LV-3004 Jelgava, Latvia; 4 Laboratory of Pharmaceutics and Phytopharmaceutical Drug Development, Faculty of Pharmaceutical Sciences, University of Kinshasa, B.P. 212, Kinshasa 012, Congo; 5 Department of Pharmacy, Faculty of Medecine and Pharmacy, University of Kisangani, B.P. 212, Kisangani 012, Congo; 6 Department of Pharmacy, Faculty of Pharmaceutical Sciences and Public Health, Official University of Bukavu, Bukavu B.P. 570, Congo; 7 Centre de Recherche et d’Innovation Technologique en Environnement et en Sciences de la Santé (CRITESS), University of Kinshasa, B.P. 212, Kinshasa 012, Congo

**Keywords:** green synthesis, *Calendula officinalis*, *Hyssopus officinalis*, silver nanoparticles, phytochemical analysis, antibacterial activity, antioxidant activity

## Abstract

Silver nanoparticles (AgNPs) biosynthesized using aqueous medical plant extracts as reducing and capping agents show multiple applicability for bacterial problems. The aim of this study was to expand the boundaries on AgNPs using a novel, low-toxicity, and cost-effective alternative and green approach to the biosynthesis of metallic NPs using *Calendula officinalis (Calendula)* and *Hyssopus officinalis (Hyssopus)* aqueous extracts. The formation of AgNPs was confirmed by transmission electron microscopy (TEM), scanning electron microscopy (SEM), and energy-dispersive spectroscopy (EDS) techniques. The effectiveness of biosynthesized AgNPs in quenching free radicals and inhibiting the growth of Gram-positive and Gram-negative microorganisms was supported by in vitro antioxidant activity assay methods and using the Kirby–Bauer disk diffusion susceptibility test, respectively. The elucidated antimicrobial and antioxidative activities of medical plant extracts were compared with data from the engineered biosynthetic AgNPs. The antimicrobial effect of engineered AgNPs against selected test cultures was found to be substantially stronger than for plant extracts used for their synthesis. The analysis of AgNPs by TEM revealed the presence of spherical-shaped nano-objects. The size distribution of AgNPs was found to be plant-type-dependent. The smaller AgNPs were obtained with *Hyssopus* extract (with a size range of 16.8 ± 5.8 nm compared to 35.7 ± 4.8 nm from *Calendula* AgNPs). The AgNPs’ presumably inherited biological functions of *Hyssopus* and *Calendula* medical plants can provide a platform to combat pathogenic bacteria in the era of multi-drug resistance.

## 1. Introduction

The expansion of multidrug-resistant (MDR) pathogens has become a global health concern during this century [1]. Indeed, the availability of MDR pathogens significantly endangers the health of both animals and consumers due to the potential risk of entering the food chain and causing severe poisoning [2]. The resistance of MDR pathogens to topical antibiotics and biocides is a result of the extensive use of antibiotics, which is the main driver leading to the accumulation of genes in bacteria, with each gene coding for resistance to a specific agent or responsible for the action of multidrug efflux pumps [3]. The lack of effective biological solutions for the treatment of MDR pathogens, along with the government directives set out in the current EU Regulation 2019/6 [4], drives researchers all over the world to investigate alternative tools to reduce this need in both human and veterinary medicine [5,6]. To date, efficient biological solutions involving active pharmaceutical ingredients (API) for the treatment of MDR pathogens and their caused infections, ensuring prolonged antibacterial activity, are still limited. It has been revealed that some of the biological molecules can be modified by covalent conjugation with polyethylene glycol (PEG) within the PEGylation process and subsequently used as adjuvants to drugs that substantially improve the solubility of the latter and decrease its immunogenicity [7]. Meanwhile, the potential utilization of phytochemicals in the drug formulations as adjuvant molecules was reported to be effective in combating MDR cancer types [8]. Given the evidence of the health-promoting benefits of *Calendula officinalis (Calendula)* and *Hyssopus officinalis (Hyssopus)*, including the substantial intrinsic antimicrobial activity against Gram-positive and Gram-negative bacteria along with superb free-radical scavenging potential [9,10,11,12], the development of new antimicrobial agents effective in treating MDR pathogens has become quite realistic.

Recent cutting-edge nanotechnology research has come with the development of plenty of nanoobjects demonstrating multiple functionalities and a range of applicability [13]. In particular, silver nanoparticles (AgNPs) attract tremendous attention from researchers due to their multiple and simultaneous mechanisms of action in combination with antimicrobial agents [14]. The ability of AgNPs to pose both unique antibacterial and antifungal effects and obvious cytotoxicity against cancer cells by destroying their ultrastructure and inducing reactive oxygen species (ROS) production and causing DNA damage, leading to eventual apoptosis or necrosis of the cells, puts them in high demand [15,16,17]. The peculiar properties of AgNPs, such as optical, electrical, mechanical, and thermal, allow them to be used both in medical and healthcare products manufacturing [18]. AgNPs could be produced by a series of methods such as physical, biological, chemical, and electrochemical photochemical, etc., with varying yields, inputs, reactions, and, as a consequence, the size and shape of AgNPs.

Green synthesis among other methods offers simplicity in operational and process conditions as its uses exclusively plant-derived metabolites and metallic salts while omitting the use of toxic reagents, catalysts, and solvents [19]. The key point in the successful synthesis of nanoparticles, however, is the availability of functional groups in bioreductants such as carboxylic, hydroxyl, and carbonyl groups that are reported to be involved in the bioreduction of Ag ions to NP along with stabilizing properties [20,21]. A variety of studies have been performed in recent years aimed at the synthesis of AgNPs using a diverse range of plant materials such as *Calotropis gigantea* [22], *Annona Squamosa* [23], *Acer oblongifolium* [24], *Cascabela thevetia* [25], *Cymbopogon citratus* [26], *Eucalyptus globulus,* and *Salvia officinalis* [27]. However, due to the relative diversity and stability of secondary metabolites, green tea leaves are most frequently reported in the literature as a natural source of reducing agents used in the biosynthesis of AgNPs [13,28]. According to HPLC analysis of the phenolic profile before and after the green synthesis of Ag and gold (Au) NPs using *Eucalyptus globulus* bark extract, Santos et al. [29] revealed that gallic acid and galloyl derivatives were primarily responsible for the reduction of Ag ions to NP. However, the importance of flavonoids, especially quercetin, as bioreductants was highlighted by Karuvantevida et al. [30]. The abundance of scientific evidence testifies to the rich bioactive composition of *Calendula* and *Hyssopus*, specifically the presence of bioactive containing vicinal diols such as quercitrin, isoquercitrin, quercetin, and chlorogenic acid [12,31]. According to these findings, these plant species could be considered a sustainable and cost-effective alternative to AgNP production. Moreover, given the intrinsic biological activities of *Calendula* and *Hyssopus* that are speculated to be inherited by synthesized AgNPs, we promoted the design of this study aimed at exploiting these widely distributed medicinal plants of *Calendula* and *Hyssopus* in the green synthesis of AgNPs.

The present paper reports the biological synthesis of silver nanoparticles using the aqueous extracts of *Hyssopus* and *Calendula* and the evaluation of AgNPs’ potential application as an antibacterial agent against various Gram-positive and Gram-negative bacteria strains with antioxidant potential.

## 2. Results and Discussion

### 2.1. Antioxidant Activity of Calendula officinalis and Hyssopus officinalis Plant AQUEOUS Extracts and Biosynthesized Silver Nanoparticles

The evidence reveals that the extracts of the *Hyssopus* and *Calendula* genus represent its abundance in chemical composition, especially antioxidants with well-documented health-promoting properties including anti-inflammatory, healing, and antiseptic [11,32]. Due to the availability of such compounds as chlorogenic, protocatechuic, ferulic, syringic, p-hydroxybenzoic, and caffeic acids, their use in the development of new drugs for the treatment of cancer and other disease has been proposed by Ghaffar and El-Elaimy [33]. In the synthesis of NPs, the presence of functional groups, especially OH and COOH, greatly promote the formation of AgNPs from AgNO_3_ by acting as reducing and stabilizing agents [34,35]. Meanwhile, the AgNPs mediated by plant extracts with intrinsic biological activities could inherit their biological functions and provide a platform to combat pathogenic bacteria in the era of multi-drug resistance [36,37].

**Figure 1:**
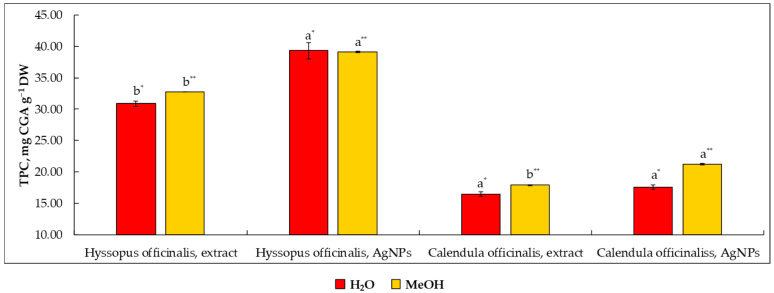
The number of total phenolics in *Hyssopus* and *Calendula* water extracts and their mediated silver nanoparticles according to the determination method (H_2_O and MeOH used as diluent). Note: Values are means ± SD of triplicates (n = 3). Means within the same plant material and diluent (H_2_O* or MeOH**) with different superscript letters (^a,b^) are significantly different (one-way ANOVA and Tukey’s test, *p* ≤ 0.05). SD—standard deviation; DW—on a dry weight basis.

The intention of this experiment was to elucidate the antioxidant activity of crude plant extracts recovered from the leaves of *Hyssopus* and inflorescences of *Calendula* along with engineered AgNPs mediated by these extracts as reducing agents (Figure 1). The obtained results indicated that both plants are rich in Total phenolic content (TPC), with *Hyssopus* leaf extract having the highest TPC content while *Calendula* had the lowest, with values corresponding to 30.90 and 16.46 mg CGA g^−1^, respectively. The observed values are in line with those of Ulewicz-Magulska and Wesolowski [38] and Andritoiu et al. [39], indicating TPC values of 35.5 and 22.7 mg of gallic acid equivalents per g^−1^ of *Hyssopus* leaves and *Calendula* flowers, respectively. It has been revealed that AgNPs mediated by plant extracts were found to possess better free-radical scavenging due to additional OH and COOH groups capped from other bioactives present in plant extracts. This observation could be additionally reinforced by Radenkovs et al. [40] who highlighted that compounds such as reducing sugars and amino and organic acids in crude extracts of plant origin can be oxidized by the Folin–Ciocalteu reagent, contributing to higher TPC values than for individual compounds or selectively purified extracts.

The observed values of TPC for AgNPs were found to be 27.2% and 6.7% higher than for crude extracts of *Hyssopus* and *Calendula*, respectively. The results are consistent with those of Salari et al. [41], indicating that the antioxidant activity of engineered AgNPs was substantially higher than the crude extracts themselves. The dilution of plant extracts in MeOH did not lead to a significant change in TPC values for plant extracts. However, better dissolution of synthesized AgNPs was observed in MeOH rather than in H_2_O. This phenomenon is explained by the amphiphilic nature of MeOH, which has more affinity to AgNPs containing OH groups than to H_2_O, thereby ensuring better uniform spatial distribution of AgNPs within the solution [42]. Negligible dissolution of AgNPs was also highlighted by Steinmetz et al. [43], demonstrating only 1.3% of dissolved AgNPs in water.

**Figure 2:**
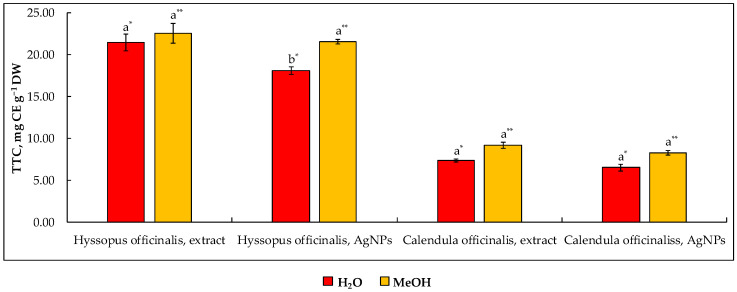
The amount of total tannins in *Hyssopus* and *Calendula* water extracts and their mediated silver nanoparticles according to the determination method (H_2_O and MeOH used as diluent). Note: Values are means ± SD of triplicates (n = 3). Means within the same plant material and diluent (H_2_O* or MeOH**) with different superscript letters (^a,b^) are significantly different (one-way ANOVA and Tukey’s test, *p* ≤ 0.05). SD—standard deviation; DW—on a dry weight basis.

Despite that astringency is a distinctive characteristic of *Calendula* flowers, the report of Verma et al. [44] emphasizes that this plant cannot be considered a source of tannins. This statement was partially confirmed by data from the current study since the concentration of total tannins (TTC) in the aqueous extract of *Calendula* inflorescence was found in a moderate amount, corresponding to 7.3 mg CE g^−1^ DW (Figure 2). As observed, no statistically significant difference was observed between the methods used for the analysis of TTC utilizing either water or MeOH as the diluent. The presence of OH groups in both hydrolyzable and condensed tannins makes these molecules extremely soluble in water. It is worth noting that a statistically similar concentration of TTC was observed in engineered AgNPs mediated by the water extract of *Calendula*. This phenomenon can be explained by the ability of tannins to form complexes not exclusively with nitrogen-containing compounds such as proteins but also with metal ions as reported by [30,45].

The concentration of TTC in water extracts of *Hyssopus* was found to be 21.4 (diluted in H_2_O) and 22.5 (diluted in MeOH) mg CE g^−1^ DW, which is 192.1% higher than that observed in *Calendula* extracts. Given the lack of data on TTC in *Hyssopus* indicating that this plant only contains a large amount of bitter and antioxidative tannins, a direct comparison of the literature data with current values is not possible. It is worth noting, however, that the content of tannins in engineered AgNPs compared to those diluted in MeOH prior to analysis was found to be statistically similar to values observed in *Calendula* extracts but 16.6% lower than that observed in AgNPs diluted in water.

**Figure 3:**
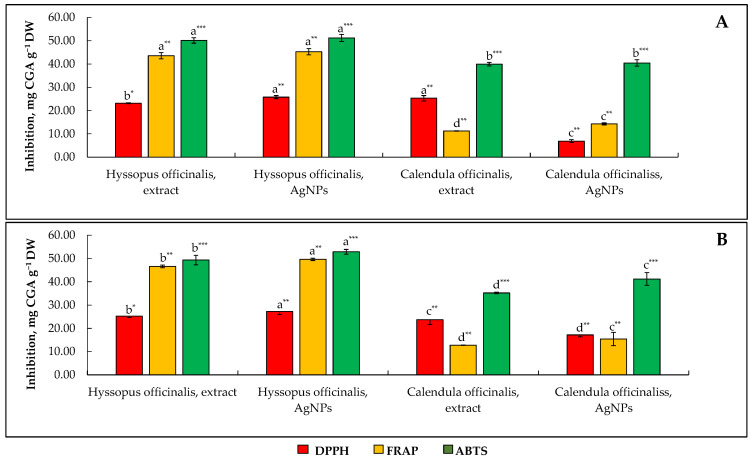
Antioxidant activity of *Hyssopus* and *Calendula* water extracts and their mediated silver nanoparticles according to the determination method, i.e., DPPH*, FRAP**, and ABTS***. Note: Values are means ± SD of triplicates (n = 3). Means within the same antioxidant activity assay method with different superscript letters (^a,b,c,d^) are significantly different (one-way ANOVA and Tukey’s test, *p* ≤ 0.05). SD—standard deviation; DW—on a dry weight basis. (**A**) Extracts and silver nanoparticles diluted in water prior to assay, (**B**) extracts and silver nanoparticles diluted in acidified 80% methanol prior to assay.

One of the most important biological effects of plant water extracts is antioxidant activity, which is closely related to antibacterial and other biological effects [46]. For these reasons, in order to evaluate the antioxidant activity of the studied *Hyssopus* and *Calendula* extracts as accurately as possible, experiments on antioxidant activity were performed in vitro using three types of antioxidant activity, i.e., ferric reducing antioxidant power (FRAP), ABTS^•+^ (2,2′-azinobis(3-ethylbenzothiazoline-6-sulphonic acid)) free-radical scavenging activity assay, and DPPH^•^ (1,1-diphenyl-2-picrylhydrazyl) free radical scavenging activity assay methods. The obtained results are presented in Figure 3.

The examination of selected plant extracts and engineered AgNPs using three in vitro spectrophotometric antioxidant activity assays demonstrated that the strongest in vitro antioxidant activity was achieved by the ABTS^•+^ method. A similar observation was made in the previous work of Balciunaitiene et al. [47], demonstrating relatively higher antioxidant activity values obtained by ABTS^•+^ for both medicinal plants and engineered AgNPs than by the other four methods. Statistically significant differences between the methods used could be explained by the mechanism by which free radicals are quenched by antioxidants. It has been reported that the ABTS^•+^ assay is based on the formation of a blue/green ABTS^•+^ chromophore, which is applicable to both hydrophilic and lipophilic natures of antioxidants, whereas the DPPH^•^ assay employs a stable free radical dissolved in organic media and is, therefore, usually used as an antioxidant activity assay for hydrophobic systems such as fat-soluble vitamins (tocopherols, tocotrienols), fatty acids, and sterols [45,48]. The ABTS^•+^ radical scavenging activity of plant extracts shows values ranging between 39.9 and 50.1 mg CGA g^−1^ DW, with *Hyssopus* having the highest value and *Calendula* the lowest. No statistically significant differences were found between H_2_O and MeOH used as the diluent for the analysis of antioxidant activity. Relatively lower but still significant values of antioxidant activity were observed when performing the analysis using the FRAP method. The antioxidant activity values varied in the range of 14.3 to 43.6 mg CGA g^−1^ DW, with *Hyssopus* having the highest value and *Calendula* the lowest. The values of the antioxidant activity of *Calendula* are inconsistent with those reported by Escher et al. [49], observing a FRAP value of 50% for EtOH extracts as 3.2 expressed as mg of ascorbic acid equivalents antiradical activity per g of raw material. The difference in values is due to the antioxidant activity being expressed in a different way.

As expected, the additional hydroxyl and carboxylic moieties inherited by AgNPs from bioactives of plant extracts ensured better radical scavenging activity of engineered nano-objects compared with crude plant extracts. This observation is in line with Shah et al. [50], reporting the availability of functional groups on the surface of AgNPs confirmed by Fourier transform infrared (FT-IR) spectroscopy. The observed ability of biogenic AgNPs to neutralize free radicals was confirmed by colorimetric assays using Rhodamine B dye under fluorescence spectrophotometry [51].

### 2.2. Structural Analysis of Silver Nanoparticles

**Figure 4:**
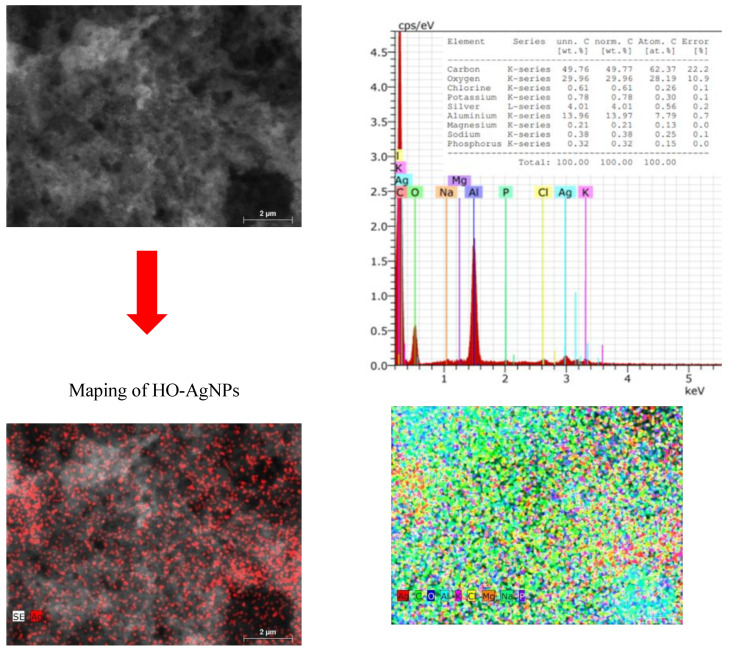
SEM images and EDS spectra of AgNPs biosynthesized using extract of *Hyssopus*.

**Figure 5:**
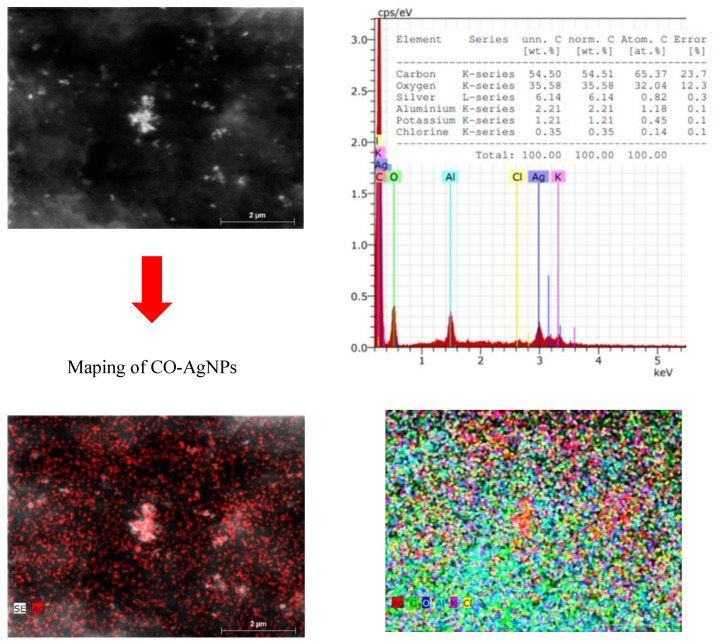
SEM images and EDS spectra of AgNPs biosynthesized using extracts of *Calendula*.

The morphology, size, shape, and chemical composition of biosynthesized HO-AgNPs and CO-AgNPs were examined using SEM-EDS and TEM techniques. SEM images and EDS spectra of AgNPs synthesized using the extract of *Hyssopus* and *Calendula* are presented in Figure 4 and Figure 5. This shows that most of the silver nanoparticles are nearly spherical in form. EDS spectra show peaks at 3.0 keV, which can be attributed to the binding energy of silver and confirm the formation of AgNPs [52]. It can be seen that the AgNPs are evenly distributed throughout the studied area (Figure 4).

The formation of silver nanoparticles is confirmed by images and the EDS spectrum. In this case, a slight agglomeration of nanoparticles is also observed. However, we can still observe that the silver nanoparticles are distributed throughout the apparent area (Figure 5).

**Figure 6:**
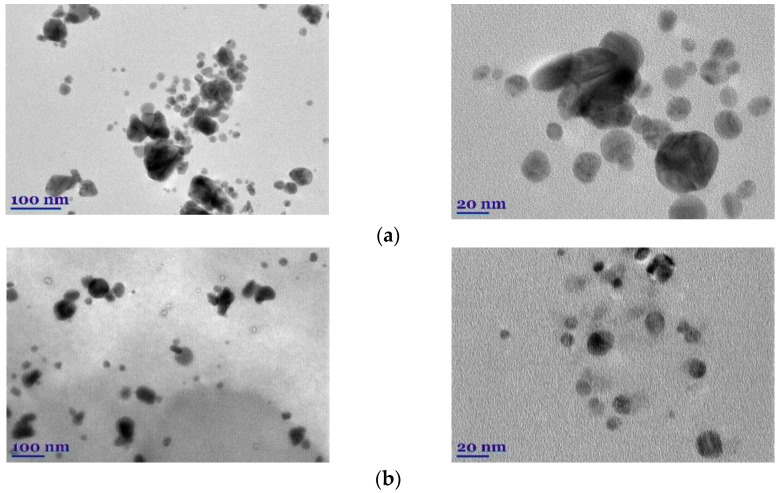
TEM images of biosynthesized CaO-AgNPs (**a**) and HyO-AgNPs (**b**).

The morphology, size, and shape of biosynthesized CaO-AgNPs and HyO-AgNPs were examined by TEM. TEM studies showed that the nanoparticles are mainly spheroidal in shape (Figure 6). We can also see that the particles do not tend to form large agglomerates. We can observe that the type of extract and its concentration affect the shape of nanoparticles. After evaluating the AgNPs’ distribution via an image application, it was found that the size of nanoparticles depends on the plant extract used for bioreduction and stabilization.

The diameter of the CaO-AgNPs was found to be in the range of 35.7 ± 4.8 nm (polydispersity of approximately 4.5). HyO-AgNPs’ diameter is lower and is in the range of 16.8 ± 5.8 nm (polydispersity of approximately 6.0).

### 2.3. Antibacterial Activity

The antibacterial activity of CaO-AgNPs and HyO-AgNPs was investigated against both Gram-negative and Gram-positive bacterial strains. From the results presented in Table 1, it can be concluded that the obtained silver nanoparticles actively interact with the bacterial membrane and disrupt their functions very actively. The aqueous extract of *Calendula* showed antimicrobial activity, but only against a few tested bacteria, *Staphylococcus aureus*, *ß*-*streptococcus,* and *Staphylococcus epidermidis*. Weak antimicrobial activity was obtained when the size of the transparent zones was 0.5 to 2.00 mm. However, *Hyssopus* extract was characterized by somewhat stronger antimicrobial activity. After synthesizing AgNPs in the extracts, stable solutions of AgNPs with high antibacterial activity were obtained, which completely inhibit various bacterial cultures. The antimicrobial activity of CaO-AgNPs was from 7.00 to 17.90 mm. Gram-positive bacterial species were characterized by somewhat stronger antimicrobial activity. The antimicrobial activity of HyO-AgNPs was from 6.00 to 16.50 mm.

The wall of Gram-positive bacteria, which is 15–80 nm thick, consists of several tens of peptidoglycan layers. This wall is not dense, so even large molecules pass through. As a result, most biologically active substances can easily enter the cells of these bacteria and affect their growth and reproduction. Gram-negative bacteria are less sensitive to the effects of antibacterial compounds because the walls of these bacteria have one or more peptidoglycan layers and an additional outer membrane consisting of lipopolysaccharides, lipoproteins, and phospholipids. Under physiological conditions, the wall has a negative charge and protects the bacterial cell from phagocytosis, the effects of enzymes, and prevents many substances from entering it.

## 3. Materials and Methods

### 3.1. Chemicals and Reagents

All commercial polyphenol standards—chlorogenic acid (CGA), gallic acid (GA), catechin, tannic acid, Folin–Ciocalteu reagent, sodium carbonate (Na_2_CO_3_), potassium persulfate (K_2_S_2_O_8_), potassium ferricyanide (K_3_Fe(CN)_6_), anhydrous ferric chloride (FeCl_3_), and phosphate-buffered saline (pH 7.4)—were purchased from Sigma-Aldrich Chemie Ltd. (Steinheim, Germany). Silver nitrate (AgNO_3_) (puriss p.a., ≥99.9%), methanol (MeOH), hydrochloric acid (HCl), 2,4,6-tripyridyl-s-triazine (TPTZ), 0.3 M acetate buffer (0.3 mol L, pH 3.6), ferric chloride hexahydrate (FeCl_3_·6H_2_O), and ferrous sulfate heptahydrate (FeSO_4_⋅7H_2_O) were purchased from Merck (Darmstadt, Germany). Ethanol (96.3% *v/v*) was obtained from Stumbras, AB (Kaunas, Lithuania). All chemicals used were of analytical grade. The ultrapure water (UPW) was produced using the reverse osmosis PureLab Flex Elga water purification system (Veolia Water Technologies, Paris, France).

### 3.2. Plant Material

Finely cut and dried *Calendula officinalis* (Švenčionių˛ vaistažolės UAB, Švenčionys, Lithuania) inflorescences and *Hyssopus officinalis* (Acorus Calamus UAB, Pakruojis, Lithuania) leaves were purchased from a public pharmacy operating in Kaunas (Lithuania). Plant material was ground to a powder to reach a Ø 0.5 mm particle size using an “IKA^®^ A11 basic” rotor mill (Staufen, Germany). Moisture content was analyzed gravimetrically with a “Precisa HA 300” (Precisa Instruments AG, Dietikon, Switzerland) moisture analyzer. Collected data (*n* = 30 of each sample) were recalculated for the absolute dry weight (DW).

### 3.3. Preparation of Plant Flowers and Leaf Extracts

A quantity of 125 g of raw material (crushed *Calendula* or *Hyssopus*) was soaked for 3 h in 70% (*v/v*) ethanol. The soaked raw material was transferred to a percolator, covered with the extractant, i.e., 70% (*v/v*) ethanol, and left to macerate for 48 h 24 °C. Then, it was percolated at a speed of 0.3 mL/min and high-concentration extract (85% of the total extract amount) was obtained. The low-concentration extract was decanted until all biologically active substances were washed from the raw material. It was evaporated using an “IKA^®^ HB 10” rotary evaporator (IKA^®^-Werke GmbH & Co. KG, Breisgau, Germany) up to 15% of the total liquid extract amount. The remaining part of the low-concentration extract was transferred to a single container with the high-concentration extract, and a liquid extract for antioxidant activity analysis was obtained. The ethanolic phase of *Calendula* and *Hyssopus* liquid extracts was evaporated at 70 °C for 4 h by a rotary evaporator (Buchi Rotavapor R-205, Buchi AG, Flawil, Switzerland) and aqueous phase extracts of yellowish color were obtained. These aqueous extracts were used as green reductants and capping agents for the biosynthesis of AgNPs. In addition, a portion of *Calendula* and *Hyssopus* aqueous extracts was lyophilized at a 0.01 mbar pressure and a condenser temperature of −85 °C using a “Zirbus lyophilizer” (Zirbus technology GmbH, Bad Grund, Germany) for morphological studies.

### 3.4. Green Synthesis of Silver Nanoparticles

A quantity of 30 mg of AgNO_3_ was dissolved in 2.5 mL UPW and mixed with 30 mL of *Calendula* or *Hyssopus* aqueous extracts under vigorous stirring at room temperature for 2 h. The mixtures were incubated at room temperature for 24 h. After that, the obtained AgNP colloids were kept in a cool, dry, and dark place.

### 3.5. Determination of Total Phenolics Content

The total phenolics content (TPC) was determined using the colorimetric Folin–Ciocalteu method [53]. The stock solutions of extracts and AgNPs were prepared from 0.2 mL aliquot of each extract and AgNPs by mixing with 1.8 mL with ultrapure water (UPW) and subsequent vortex mixing and ultrasonication for 1 and 5 min at room (22 ± 1 °C) temperature, respectively. Since the dissolution of AgNPs can vary depending on the AgNPs’ surface charge and capping agent used, resulting in the underestimation of antiradical activity, under a separate trial, both the prepared extracts and AgNPs were dissolved in acidified 80% MeOH (MeOH:H_2_O:hydrochloric acid ratio 89:19:0.1 *v/v/v*) for comparative purposes. Afterward, a 0.5 mL aliquot of each sample (from stock) or standard or blank (UPW) was mixed with 2.5 mL of a 10-fold diluted Folin–Ciocalteu reagent and 2.0 mL of 7.5% Na_2_CO_3_ with subsequent vortex mixing for 1 min. The prepared solutions were incubated for 30 min at room temperature. Finally, the absorbance was measured at 760 nm using a UV-1800–Visible Spectrophotometer (Shimadzu Corp., Kyoto, Japan). The results were expressed as chlorogenic acid (CGA) equivalents per g of raw material (mg CGA g^−1^).

### 3.6. Determination of Total Tannins Content

The concentration of the total tannins (TTC) was determined following the methodology provided by Graham [54], with some modifications. The stock solutions of extracts and AgNPs were prepared from 0.2 mL aliquots of each extract and AgNPs by mixing them with either 1.8 mL UPW or acidified 80% MeOH (MeOH:H_2_O:hydrochloric acid ratio 89:19:0.1 *v/v/v*) and subsequent vortex mixing and ultrasonication for 1 and 5 min at room (22 ± 1 °C) temperature, respectively. Afterward, a 0.5 mL aliquot of each sample (from stock) or standard or blank (UPW) was transferred to 15 mL tubes and mixed with 1.0 mL 1% K_3_Fe(CN)_6_ and 1% FeCl_3_ in 0.1 N HCl. The obtained solution was adjusted with UPW to the final volume 10.0 mL, mixed well, and allowed to stand for 10 min at 22 ± 1 °C in the dark. The absorbance was measured at 720 nm using a UV-1800–Visible Spectrophotometer (Shimadzu Corp., Kyoto, Japan). The actual tannin concentrations were calculated based on the optical absorbance values obtained for the standard solutions. The results were expressed as mg catechin (CE) equivalents per g of raw material (mg CE g^−1^).

### 3.7. Evaluation of Antioxidant Activity

#### 3.7.1. DPPH^•^ Radical Scavenging Activity

The DPPH^•^ assay was conducted based on the method described by Radenkovs et al. [55] with slight modification. Briefly, a 0.15 mL aliquot of each sample or standard or blank (UPW) was mixed with 2.85 mL of DPPH^•^−EtOH solution (0.039 g DPPH^•^ in 1 L EtOH). The prepared solution was reacted for 30 min at 22 ± 1 °C in the dark. The absorbance of the extracts and AgNPs was measured at 0 and 30 min at a wavelength of 517 nm (A517) using a UV-1800–Visible Spectrophotometer (Shimadzu Corp., Kyoto, Japan). The DPPH^•^ scavenging activity was calculated using a calibration curve of the standard and expressed as mg of CGA equivalent antiradical activity per g of raw material (mg CGA g^−1^).

#### 3.7.2. The Ferric Reducing Antioxidant Power (FRAP) Assay

Ferric reducing antioxidant power (FRAP) was determined following the protocol described by Radenkovs et al. [55]. The FRAP reagent was prepared daily from a 100 mL acetate buffer (0.3 mol L^−1^; pH 3.6), a 2,4,6-tripyridyl-s-trizine (TPTZ) solution in 10 mmol L^−1^ of HCl, and FeCl_3_ (20 mmol L^−1^). The three solutions were mixed together at a ratio of 10:1:1 (*v/v/v*), respectively, and then heated at 37 °C. Then, a 0.15 mL aliquot of each sample or standard was mixed with the 2.85 mL FRAP reagent and vortex mixed for 1 min. The resulting absorbance was measured at a wavelength of 593 nm after 10 min against a blank sample (UPW), which was used as the reference. The assay was performed using a UV-1800–Visible Spectrophotometer (Shimadzu Corp., Kyoto, Japan) The results were calculated using a calibration curve of the standard and expressed as mg CGA mL^−1^.

#### 3.7.3. ABTS^•+^ Radical Cation Scavenging Activity

The scavenging activity of ABTS^•+^ was determined according to the method of Du et al. [56] with some modifications. The synthetic radical ABTS^•+^ was prepared by mixing 0.15 mL of the 7.4 mM ABTS^•+^ solution with 0.15 mL of 2.6 mM potassium persulfate (K_2_S_2_O_8_). The prepared solution was allowed to react for 16 h at room temperature in the dark. The ABTS^•+^ solution was then diluted with phosphate-buffered saline (PBS) with an adjusted pH of 7.4 to an absorbance of 0.70 ± 0.02 at 734 nm. An aliquot of 0.15 mL of each sample, standard, or blank (PBS) was added to 2.85 mL of diluted ABTS^•+^ and left to react for 10 min at 22 ± 1 °C in the dark, and absorbance at 734 nm was measured using a UV-1800–Visible Spectrophotometer (Shimadzu Corp., Kyoto, Japan). The results were calculated using a calibration curve of the standard expressed as mg CGA g^−1^.

### 3.8. Scanning Electron Microscopy (SEM) Analysis

The particle size and structures were studied from the images obtained by SEM FEI Quanta 200 FEG (FEI Company, Hillsboro, OR, USA) The samples were examined in low-vacuum mode operating at 20.0 kV using an LDF detector. The content of AgNPs and the chemical analysis of nanocomposites were performed by the energy dispersive spectroscopy SEM/EDS technique with a BruckerXFlash 4030 detector (an accelerating voltage of 10 kV with a distance between the bottom of the objective lens and the object of 10 mm).

### 3.9. Transmission Electron Microscopy (TEM)

Tecnai G2 F20 X-TWIN (FEI, USA) examined the size of the synthesized nanoparticles under a transmission electron microscope (TEM). For the TEM experiment, the diluted samples were deposited drop-wise onto carbon-coated copper TEM grids. A Field Emission Gun electron source was used, of which the accelerating voltage was 200 kV. The resolution of the microscope ranged from 0.8 to 1.0 nm. The EDAX spectrometer with the r-TEM detector and 11 MPix ORIUS SC1000B (Gatan Inc., Pleaston, CA, USA) CCD camera was used. The spot/linear resolution was 0.25/0.102 nm.

### 3.10. Antibacterial Assay

The antibacterial activity of synthesized silver nanoparticles in the plant extract was evaluated in vitro using the Agar diffusion test. Selected Gram-positive bacteria strains (*Proteus vulgaris* ATCC 8427, *Staphylococcus aureus* ATCC 25923, *ß-streptococcus* ATCC 19563, and *Bacillus cereus* ATCC 6633) and Gram-negative bacteria strains (*Klebsiella pneumoniae* ATCC 13883, *Proteus mirabilis* ATCC 29906, *Escherichia coli* ATCC 25922, and *Pseudomonas aeruginosa* ATCC 27853), *Enterococcus faecalis* ATCC 51299, *Bacillus cereus* ATCC 11778, and the fungi *Candida albicans* ATCC 32354 were used for analysis. For this purpose, the 0.5 McFarland unit density suspension (~10^8^ CFU/mL) of the bacterial strain was inoculated onto the cooled Mueller Hinton Agar (Oxoid, Basingstoke, UK) using sterile cotton swabs. Wells (Ø = 6 mm) were punched in the agar and filled with 50 μL of extracts. Agar plates were incubated at 37 °C for 24 h, and inhibition zones were measured and tabulated [37,57].

### 3.11. Statistical Analysis

Experiments were carried out in triplicate. Means and standard deviations were calculated with STATISTICA 10 (StatSoft, Inc., Tulsa, OK, USA) software. A one-way analysis of variance (ANOVA) with the post hoc Tukey’s HSD test was employed for statistical analysis. Differences were significant at *p* < 0.05.

## 4. Conclusions

The dispersed AgNPs were synthesized by the green, sustainable, and eco-friendly method using medical plant extracts of *Hyssopus* and *Calendula* as a capping and reducing agent. The presented results confirmed that the morphology and properties of silver nanoparticles are influenced by the selected plant. The size of green AgNPs mediated by *Hyssopus* was found to be 16.8 ± 5.8 nm. However, the size range of AgNPs obtained by *Calendula* was found to be significantly larger at 35.7 ± 4.8 nm. Phenolic compounds, tannins, flavonoids, and other phytochemicals found in plant extracts and capped silver nanoparticles are responsible for their biological activity. The biosynthesized green silver nanoparticles exerted prominent antioxidant properties and antibacterial potency against all Gram-positive and Gram-negative bacteria strains. The antioxidant activity values varied in the range of 14.3 to 43.6 mg CGA g^−1^ DW, with *Hyssopus* having the highest value and *Calendula* the lowest. The antimicrobial activity of CaO-AgNPs and HyO-AgNPs was obtained at 7.00 to 17.90 mm and 6.00 to 16.50 mm, respectively.

## Figures and Tables

**Table 1:** The antibacterial activity of CaO-AgNPs and HyO-AgNPs.

Reference (Standard) Cultures of Microorganisms	*Calendula*	CaO-AgNPs	*Hyssopus*	HyO-AgNPs
*Staphylococcus aureus*	1.50 ± 0.10	15.10 ± 0.10	1.20 ± 0.15	14.50 ± 0.15
*ß- streptococcus*	2.00 ± 0.10	17.9 ± 0.10	1.70 ± 0.10	16.50 ± 0.50
*Staphylococcus epidermidis*	0.5 ± 0.55	12.50 ± 0.10	2.05 ± 0.25	16.50 ± 0.10
*Escherichia coli*	0.00 ± 0.10	10.50 ± 0.35	0.00 ± 0.10	12.50 ± 0.10
*Klebsiella pneumoniae*	0.00 ± 0.00	10.00 ± 0.10	0.00 ± 0.00	10.40 ± 0.10
*Pseudomonas aeruginosa*	0.00 ± 0.00	10.00 ± 0.10	0.00 ± 0.00	10.00 ± 0.90
*Proteus vulgaris*	0.00 ± 0.00	12.50 ± 0.04	0.00 ± 0.00	11.40 ± 0.00
*Bacillus cereus*	0.00 ± 0.05	11.80 ± 0.55	0.50 ± 0.10	9.50 ± 0.10
*Enterococcus faecalis*	0.00 ± 0.00	10.40 ± 0.50	0.50 ± 0.10	10.00 ± 0.50
*Candida albicans*	0.00 ± 0.45	9.20 ± 0.10	0.9 ± 0.40	6.00 ± 0.10

## Data Availability

All data generated during this study are included in this article.

## References

[1] Pérez-Rodríguez F., Mercanoglu Taban B. (2019). A state-of-art review on multi-drug resistant pathogens in foods of animal origin: Risk factors and mitigation strategies. Front. Microbiol..

[2] Doyle M.E. (2015). Multidrug-resistant pathogens in the food supply. Foodborne Pathog. Dis..

[3] Nikaido H. (2009). Multidrug resistance in bacteria. Annu. Rev. Biochem..

[4] European Union (2019). No. 2019/6: Regulation of the European Parliament and of the Council of 11 December 2018 on Veterinary Medicinal Products and Repealing Directive 2001/82/EC.

[5] Pacios O., Blasco L., Bleriot I., Fernandez-Garcia L., González Bardanca M., Ambroa A., López M., Bou G., Tomás M. (2020). Strategies to combat multidrug-resistant and persistent infectious diseases. Antibiotics.

[6] Olofsson T.C., Butler E., Lindholm C., Nilson B., Michanek P., Vásquez A. (2016). Fighting off wound pathogens in horses with honeybee lactic acid bacteria. Curr. Microbiol..

[7] Sun J., Zhao R., Zeng J., Li G., Li X. (2010). Characterization of destrins with different dextrose equivalents. Molecules.

[8] Tinoush B., Shirdel I., Wink M. (2020). Phytochemicals: Potential lead molecules for MDR reversal. Front. Pharmacol..

[9] Efstratiou E., Hussain A.I., Nigam P.S., Moore J.E., Ayub M.A., Rao J.R. (2012). Antimicrobial activity of *Calendula officinalis* petal extracts against fungi, as well as Gram-negative and Gram-positive clinical pathogens. Complement. Ther. Clin. Pract..

[10] Faria R.L., Cardoso L.M.L., Akisue G., Pereira C.A., Junqueira J.C., Jorge A.O.C., Santos Júnior P.V. (2011). Antimicrobial activity of *Calendula officinalis*, *Camellia sinensis* and chlorhexidine against the adherence of microorganisms to sutures after extraction of unerupted third molars. J. Appl. Oral Sci..

[11] Fatemeh F., Sanaz H. (2011). A review on *Hyssopus officinalis* L.: Composition and biological activities. Afr. J. Pharm. Pharmacol..

[12] Vlase L., Benedec D., Hanganu D., Damian G., Csillag I., Sevastre B., Mot A.C., Silaghi-Dumitrescu R., Tilea I. (2014). Evaluation of antioxidant and antimicrobial activities and phenolic profile for *Hyssopus officinalis*, *Ocimum basilicum* and *Teucrium chamaedrys*. Molecules.

[13] Radenkovs V., Juhnevica-Radenkova K., Jakovlevs D., Zikmanis P., Galina D., Valdovska A. (2022). The release of non-extractable ferulic acid from cereal by-products by enzyme-assisted hydrolysis for possible utilization in green synthesis of silver nanoparticles. Nanomaterials.

[14] Bruna T., Maldonado-Bravo F., Jara P., Caro N. (2021). Silver nanoparticles and their antibacterial applications. Int. J. Mol. Sci..

[15] Arenas-Chávez C.A., de Hollanda L.M., Arce-Esquivel A.A., Alvarez-Risco A., Del-Aguila-Arcentales S., Yáñez J.A., Vera-Gonzales C. (2022). Antibacterial and antifungal activity of functionalized cotton fabric with nanocomposite based on silver nanoparticles and carboxymethyl chitosan. Processes.

[16] Gibała A., Żeliszewska P., Gosiewski T., Krawczyk A., Duraczyńska D., Szaleniec J., Szaleniec M., Oćwieja M. (2021). Antibacterial and antifungal properties of silver nanoparticles—Effect of a surface-stabilizing agent. Biomolecules.

[17] Xu L., Yi-Yi W., Huang J., Chun-Yuan C., Zhen-Xing W., Xie H. (2020). Silver nanoparticles: Synthesis, medical applications and biosafety. Theranostics.

[18] Zhang X., Liu Z., Shen W., Gurunathan S. (2016). Silver nanoparticles: Synthesis, characterization, properties, applications, and therapeutic approaches. Int. J. Mol. Sci..

[19] Rolim W.R., Pelegrino M.T., de Araújo Lima B., Ferraz L.S., Costa F.N., Bernardes J.S., Rodigues T., Brocchi M., Seabra A.B. (2019). Green tea extract mediated biogenic synthesis of silver nanoparticles: Characterization, cytotoxicity evaluation and antibacterial activity. Appl. Surf. Sci..

[20] Jain A.S., Pawar P.S., Sarkar A., Junnuthula V., Dyawanapelly S. (2021). Bionanofactories for green synthesis of silver nanoparticles: Toward antimicrobial applications. Int. J. Mol. Sci..

[21] Matur M., Madhyastha H., Shruthi T., Madhyastha R., Srinivas S., Navya P., Daima H.K. (2020). Engineering bioactive surfaces on nanoparticles and their biological interactions. Sci. Rep..

[22] Kemala P., Idroes R., Khairan K., Ramli M., Jalil Z., Idroes G.M., Tallei T.E., Helwani Z., Safitri E., Iqhrammullah M. (2022). Green Synthesis and Antimicrobial Activities of Silver Nanoparticles Using *Calotropis gigantea* from Ie Seu-Um Geothermal Area, Aceh Province, Indonesia. Molecules.

[23] Malik M., Iqbal M.A., Malik M., Raza M.A., Shahid W., Choi J.R., Pham P.V. (2022). Biosynthesis and characterizations of silver nanoparticles from *Annona squamosa* leaf and fruit extracts for size-dependent biomedical applications. Nanomaterials.

[24] Naveed M., Bukhari B., Aziz T., Zaib S., Mansoor M.A., Khan A.A., Shahzad M., Dablool A.S., Alruways M.W., Almalki A.A. (2022). Green synthesis of silver nanoparticles using the plant extract of *Acer oblongifolium* and study of its antibacterial and antiproliferative activity via mathematical approaches. Molecules.

[25] Khan A.N., Ali Aldowairy N.N., Saad Alorfi H.S., Aslam M., Bawazir W.A., Hameed A., Soomro M.T. (2022). Excellent antimicrobial, antioxidant, and catalytic activities of medicinal plant aqueous leaf extract derived silver nanoparticles. Processes.

[26] Rakib-Uz-Zaman S., Hoque Apu E., Muntasir M.N., Mowna S.A., Khanom M.G., Jahan S.S., Akter N., Khan M.A.R., Shuborna N.S., Shams S.M. (2022). Biosynthesis of silver nanoparticles from *Cymbopogon citratus* leaf extract and evaluation of their antimicrobial properties. Challenges.

[27] Balčiūnaitienė A., Liaudanskas M., Puzerytė V., Viškelis J., Janulis V., Viškelis P., Griškonis E., Jankauskaitė V. (2022). *Eucalyptus globulus* and *Salvia officinalis* extracts mediated green synthesis of silver nanoparticles and their application as an antioxidant and antimicrobial agent. Plants.

[28] Widatalla H.A., Yassin L.F., Alrasheid A.A., Ahmed S.A.R., Widdatallah M.O., Eltilib S.H., Mohamed A.A. (2022). Green synthesis of silver nanoparticles using green tea leaf extract, characterization and evaluation of antimicrobial activity. Nanoscale Adv..

[29] Santos S.A., Pinto R.J., Rocha S.M., Marques P.A., Neto C.P., Silvestre A.J., Freire C.S. (2014). Unveiling the chemistry behind the green synthesis of metal nanoparticles. ChemSusChem.

[30] Adamczyk B., Simon J., Kitunen V., Adamczyk S., Smolander A. (2017). Tannins and their complex interaction with different organic nitrogen compounds and enzymes: Old paradigms versus recent advances. ChemistryOpen.

[31] Ak G., Zengin G., Sinan K.I., Mahomoodally M.F., Picot-Allain M.C.N., Cakır O., Bensari S., Yılmaz M.A., Gallo M., Montesano D. (2020). A comparative bio-evaluation and chemical profiles of *Calendula officinalis* L. extracts prepared via different extraction techniques. Appl. Sci..

[32] Butnariu M., Coradini C.Z. (2012). Evaluation of biologically active compounds from *Calendula officinalis* flowers using spectrophotometry. Chem. Cent. J..

[33] Ibrahim H.M., Ghaffar F.R.A., El-Elaimy I.A., Gouida M.S. (2018). Antitumor and immune-modulatory efficacy of dual-treatment based on levamisole and/or taurine in Ehrlich ascites carcinoma-bearing mice. Biomed. Pharmacother..

[34] Dorjnamjin D., Ariunaa M., Shim Y.K. (2008). Synthesis of silver nanoparticles using hydroxyl functionalized ionic liquids and their antimicrobial activity. Int. J. Mol. Sci..

[35] Sharma D., Kanchi S., Bisetty K. (2019). Biogenic synthesis of nanoparticles: A review. Arab. J. Chem..

[36] Khan S.A., Shahid S., Lee C. (2020). Green synthesis of gold and silver nanoparticles using leaf extract of *Clerodendrum inerme*; characterization, antimicrobial, and antioxidant activities. Biomolecules.

[37] Balčiūnaitienė A., Štreimikytė P., Puzerytė V., Viškelis J., Štreimikytė-Mockeliūnė Ž., Maželienė Ž., Sakalauskienė V., Viškelis P. (2022). Antimicrobial activities against opportunistic pathogenic bacteria using green synthesized silver nanoparticles in Plant and Lichen Enzyme-Assisted Extracts. Plants.

[38] Ulewicz-Magulska B., Wesolowski M. (2019). Total phenolic contents and antioxidant potential of herbs used for medical and culinary purposes. Plant Foods Hum. Nutr..

[39] Andritoiu C.V., Andriescu C.E., Ibanescu C., Lungu C., Ivanescu B., Vlase L., Havarneanu C., Popa M. (2020). Effects and characterization of some topical ointments based on vegetal extracts on incision, excision, and thermal wound models. Molecules.

[40] Heyman H.M., Senejoux F., Seibert I., Klimkait T., Maharaj V.J., Meyer J.J.M. (2015). Identification of anti-HIV active dicaffeoylquinic-and tricaffeoylquinic acids in *Helichrysum populifolium* by NMR-based metabolomic guided fractionation. Fitoterapia.

[41] Salari S., Esmaeilzadeh Bahabadi S., Samzadeh-Kermani A., Yosefzaei F. (2019). In-vitro evaluation of antioxidant and antibacterial potential of green synthesized silver nanoparticles using *Prosopis farcta* fruit extract. Iran. J. Pharm. Res..

[42] Ribeiro A.I., Modic M., Cvelbar U., Dinescu G., Mitu B., Nikiforov A., Leys C., Kuchakova I., De Vrieze M., Felgueiras H.P. (2020). Effect of dispersion solvent on the deposition of PVP-silver nanoparticles onto DBD plasma-treated polyamide 6, 6 fabric and its antimicrobial efficiency. Nanomaterials.

[43] Steinmetz L., Geers C., Balog S., Bonmarin M., Rodriguez-Lorenzo L., Taladriz-Blanco P., Rothen-Rutishauser B., Petri-Fink A. (2020). A comparative study of silver nanoparticle dissolution under physiological conditions. Nanoscale Adv..

[44] Verma P.K., Raina R., Agarwal S., Kaur H. (2018). Phytochemical ingredients and Pharmacological potential of *Calendula officinalis* Linn. Pharm. Biomed. Res..

[45] Pucci C., Martinelli C., De Pasquale D., Battaglini M., di Leo N., Degl’Innocenti A., Belenli Gümüş M., Drago F., Ciofani G. (2022). Tannic acid–iron complex-based nanoparticles as a novel tool against oxidative stress. ACS Appl. Mater. Interfaces.

[46] Cushnie T.T., Lamb A.J. (2005). Detection of galangin-induced cytoplasmic membrane damage in *Staphylococcus aureus* by measuring potassium loss. J. Ethnopharmacol..

[47] Balciunaitiene A., Viskelis P., Viskelis J., Streimikyte P., Liaudanskas M., Bartkiene E., Zavistanaviciute P., Zokaityte E., Starkute V., Ruzauskas M. (2021). Green synthesis of silver nanoparticles using extract of *Artemisia absinthium* L., *Humulus lupulus* L. and *Thymus vulgaris* L., physico-chemical characterization, antimicrobial and antioxidant activity. Processes.

[48] Prevc T., Šegatin N., Ulrih N.P., Cigić B. (2013). DPPH assay of vegetable oils and model antioxidants in protic and aprotic solvents. Talanta.

[49] Bragueto Escher G., Cardoso Borges L.D.C., Sousa Santos J., Mendanha Cruz T., Boscacci Marques M., Vieira do Carmo M.A., Azevedo L., Furtado M.M., Sant’Ana A.S., Wen M. (2019). From the field to the pot: Phytochemical and functional analyses of *Calendula officinalis* L. flower for incorporation in an organic yogurt. Antioxidants.

[50] Steinfeld B., Scott J., Vilander G., Marx L., Quirk M., Lindberg J., Koerner K. (2015). The role of lean process improvement in implementation of evidence-based practices in behavioral health care. J. Behav. Health Serv. Res..

[51] Perera K.M.K.G., Kuruppu K.A.S.S., Chamara A.M.R., Thiripuranathar G. (2020). Characterization of spherical Ag nanoparticles synthesized from the agricultural wastes of *Garcinia mangostana* and *Nephelium lappaceum* and their applications as a photo catalyzer and fluorescence quencher. SN Appl. Sci..

[52] Guibal E., Cambe S., Bayle S., Taulemesse J., Vincent T. (2013). Silver/chitosan/cellulose fibers foam composites: From synthesis to antibacterial properties. J. Colloid Interface Sci..

[53] Singleton V.L., Orthofer R., Lamuela-Raventós R.M. (1999). Analysis of total phenols and other oxidation substrates and antioxidants by means of Folin-Ciocalteu reagent. Meth Enzym..

[54] Graham W., Rose A. (1938). Tannins and non-tannins of the barks of some eastern canadian conifers, particularly white spruce. Can. J. Res..

[55] Radenkovs V., Püssa T., Juhnevica-Radenkova K., Anton D., Seglina D. (2018). Phytochemical characterization and antimicrobial evaluation of young leaf/shoot and press cake extracts from *Hippophae rhamnoides* L.. Food Biosci..

[56] Du H., Wu J., Li H., Zhong P., Xu Y., Li C., Ji K., Wang L. (2013). Polyphenols and triterpenes from Chaenomeles fruits: Chemical analysis and antioxidant activities assessment. Food Chem..

[57] Streimikyte P., Kailiuviene J., Mazoniene E., Puzeryte V., Urbonaviciene D., Balciunaitiene A., Liapman T.D., Laureckas Z., Viskelis P., Viskelis J. (2022). The biochemical alteration of enzymatically hydrolysed and spontaneously fermented oat flour and its impact on pathogenic bacteria. Foods.

